# Daily versus intermittent iron supplementation in pregnant women

**DOI:** 10.1186/1756-0500-4-444

**Published:** 2011-10-25

**Authors:** Zinatossadat Bouzari, Zahra Basirat, Mahtab Zeinal Zadeh, Shahla Yazdani Cherati, Maryam Didehdar Ardebil, Maedeh Mohammadnetaj, Shahnaz Barat

**Affiliations:** 1 Department of Obstruction & Gynecology, Babol University of Medical Science, Babol, Iran; 2 Stem Cell Research Center, Babol University of Medical Science, Babol, Iran; 3 Department of Obstruction & Gynecology, Babol University of Medical Science, Babol, Iran; 4 Azad Islamic University, Sarab, Iran; 5 Department of Psychology, Panjab University, India; 6 Mazandaran University, Iran; 7 Department of Obstruction & Gynecology, Babol University of Medical Science, Babol, Iran

## Abstract

**Background:**

The purpose of this study was compare of daily iron supplementation in three time frames- daily, weekly and three time weekly supplementation in preventing anemia in healthy pregnant women.

**Method:**

The present study was a prospective simply randomized clinical trial. During January 2006- January 2008, 150 healthy pregnant women without anemia, in their 16th week of pregnancy were randomly allocated into three equal groups. The first group (n = 50) received a 50 mg-ferrous sulfate tablet daily, second group (n = 50) received a 50 mg-ferrous sulfate tablet three times a week, and the third group (n = 50) received two 50 mg-ferrous sulfate tablets (100 mg) weekly, respectively for 12 consecutive weeks. Serum hemoglobin, ferritin, and iron were measured before and after the supplementation. Paired *t *and ANOVA tests were used as appropriated.

**Results:**

There were no significant differences between the pre- and post-treatment hemoglobin levels with iron supplementation in the three group (P = 0.518, P = 0.276, respectively). The mean serum iron level before and after treatment with iron supplementation in the three groups was not statistically significant (P = 0.962, P = 0.970, respectively). Although the mean serum ferritin level before and after treatment with iron supplementation was statistically significant in the three groups, no significant differences were found comparing the three groups (P = 0.827, P = 0.635 respectively).

**Conclusions:**

This results suggested, three times a week or weekly iron supplementation is as effective as daily supplementation for healthy pregnant women without anemia.

**Trial Registration:**

ISRCTN: IRCT201101093820N1

## Background

Iron requirements increase during pregnancy [1,2]. And this requirement may lead to anemia in pregnant women [3,4]. Lower hemoglobin cut off is 11.0 g/dL in the first and last trimester and 10.5 g/dL in the second trimester. Therefore, any level below 10.5 g/dL should be considered as anemia [5]. Iron consumption for pregnant women is undesirable, because of the side effects. The probable cause is the effect of oxidative stress of high doses of Iron, which leads to gastrointestinal intolerance [3]. As gut mucosal turnover rates is about three days, administering iron during these days may lead to lower iron absorption. Periodic iron supplementation may let the mucosa to heal and gets better iron absorption [6-8]. Previous studies reported, continuous administration of oral iron impairs the absorption of a subsequent iron dose [9]. A few experimental studies demonstrated that alternative efficacious iron supplementation regimens might reduce the undesirable side-effects [10]. Significant equality and reduced side effects have been reported in several epidemiological studies in comparing the weekly prescription of iron with daily supplementation [3-7]. In the other studies, there was not found difference between daily and twice-weekly iron supplementation regimens in preventing iron deficiency anemia in children, non-pregnant women, and pregnant women in their early trimesters [12-14]. And in another study, intermittent regimen was shown to be superior to daily regimen [3-15]. In human beings, gut mucosal turnover occurs every 3 days. Thus, weekly rather than daily administration of iron has been proposed as a safe, beneficent, and cost-effective method to prevent and alleviate anemia in pregnant women [12-17]. As mentioned above there are different study which studied effect of iron separately in daily use and weekly and three times. Thus authors aimed to compare the effects of daily, three times a week, and weekly iron supplementation regimens on hematological markers in pregnant women without anemia.

## Methods

The present study was a prospective simply randomized clinical trial conducted during January 2006-January 2008 in six health centers affiliated to Babol University of Medical Sciences northern Iran. The centers were selected through a cluster randomization out of the 40 centers affiliated to the University. Serum hemoglobin (Hycel cell counter, France), serum ferritin (radioimmunoassay technique, Genesis factory, USA), and serum iron (Pars azmoon, Iran) levels were measured for all pregnant women who attended the antenatal clinics in their 14th week of gestation. Women with hemoglobin levels less than 11 g/dL, chronic hematological disorders (thalassemia, etc.), and multiple pregnancies were excluded from the study.

Hundred fifty pregnant women without exclusion criteria were enrolled in the study in their 16 th week of gestation. A written informed consent was obtained from all the participants. The Ethics Committee of the University approved the study protocol. The women were divided to three equal groups. All the groups were matched in terms of age, numbers of pregnant, income, and education. The first group (n = 50) received a 50 mg-ferrous sulfate tablet (Darou Pakhsh, Iran) daily, the second group (n = 50) received a 50 mg-ferrous sulfate tablet three times a week, and the third group (n = 50) received two 50 mg-ferrous sulfate tablets (100 mg) weekly, respectively.

Iron supplementation was prescribed in 16th week of gestation and was continued according to the mentioned regimens for 12 weeks (until 28th weeks of gestation). Serum hemoglobin, serum ferritin, and serum iron levels were measured for all the participants at the end of the trial period. And patients who were diagnosed as having anemia were treated according to the standard protocol.

### Statistical analyses

Data were recorded on a pre-designed form. All of the entries were checked twice. Collected data were analyzed using SPSS software version 13. Serum sample levels of hemoglobin, iron, and ferritin before and after treatment were evaluated using paired *t *test. ANOVA was used to compare the mean values of serum hemoglobin, ferritin, and iron between the three groups. P value<0.05 considered as statistically significant.

## Results

Totally 150 participate, enrolled in the study, among total sample eight women were excluded because of abortion, premature labor, and immigration. Therefore the study continued with 50 pregnant women in daily regimen, 43 in three-time a week regimen, and 49 in weekly regimen groups. There were not reported any side effects related to iron consumption such as nausea, vomiting, and bowel disturbances.

Serum levels of hemoglobin, iron, and ferritin before and after treatment in the three supplementation regimens are shown in (Table 1).

**Table 1:** Serum levels of hemoglobin, iron, and ferritin before and after the three supplementation regimens

		Daily (n = 50)	P value	Three-time a week (n = 43)	P value	Weekly (n = 49)	P value
Hemoglobin(g/dL, mean ± SD)	Before supplementation	12/44 ± 0/99	P<0/001*	12/62 ± 0/78	P = 0/541	12/53 ± 0/77	P<0/001*

	After supplementation	11/084 ± 0/82		14/03 ± 14/68		11/62 ± 0/82	

Serum iron (μg/dl, mean ± SD)	Before supplementation	96/74 ± 34/31	P = 0/948	94/21 ± 31/14	P = 0/516	97/28 ± 31/65	P = 0/942

	After supplementation	97/14 ± 40/06		99/90 ± 53/33		96/57 ± 62/62	

Serum ferritin (μg/L, mean ± SD)	Before supplementation	59/66 ± 45/32	P = 0/005*	58/82 ± 33/6	P = 0/00*	56/53 ± 33/33	P = 0/002*

	After supplementation	39/90 ± 4/51		32/86 ± 32/78		37/87 ± 34/95	

According to Table 1, the mean serum hemoglobin and ferritin levels were statistically different in the first and third groups but in the second group only ferritin level changes was significant. There were no significant differences between the serum hemoglobin levels before and after supplementation with iron in the three group (P = 0.518, P = 0.276, respectively, (Table 2).

**Table 2:** Comparison of serum hemoglobin, iron, and ferritin levels before and after the three supplementation regimens

	Daily (mean ± SD)		Three times a week (mean ± SD)	Weekly (mean ± SD)	P value
Hemoglobin level(g/dL)	Before supplementation	12/44 ± 0/99	12/62 ± 0/78	12/53 ± 0/77	0.518

	After supplementation	11/084 ± 0/82	14/03 ± 14/68	11/62 ± 0/82	0.276

Serum iron level(μg/dl)	Before supplementation	96/74 ± 34/31	94/21 ± 31/14	97/28 ± 31/65	0.962

	After supplementation	97/14 ± 40/06	99/90 ± 53/33	96/57 ± 62/62	0.970

Serum ferritin level (μg/L)'	Before supplementation	59/66 ± 45/32	58/82 ± 33/6	56/53 ± 33/33	0.827

	After supplementation	39/90 ± 4/51	32/86 ± 32/78	37/87 ± 34/95	0.635

The mean serum iron level before and after supplementation with iron in the three groups was

not statistically significant (P = 0.962, P = 0.970, respectively, (Table 2) Although the mean serum ferritin level before and after supplementation with iron was statistically significant in the three groups, no significant differences were found comparing the three groups (P = 0.827, P = 0.635 respectively, (Table 2).

## Discussion

In the current study, Authors found daily, three times a week, and weekly iron supplementation regimens were comparably effective in pregnant women without anemia. Serum ferritin and hemoglobin levels decreased after 12 weeks of supplementation. Serum ferritin decreased after supplementation with all three regimens. Because of more iron need during that period. However such decrease was not statistically significant.

Mukhopadhyay and colleagues compared daily consumption of 100 mg elemental iron (n = 55) with weekly consumption of 200 mg elemental iron (n = 56) in India. They found no significant difference in serum hemoglobin levels after 17 weeks of supplementation between their two groups [3]. In another study in Indonesia, Muslimatun and colleagues compared the effects of daily prescription of 60 mg elemental iron (n = 123) with weekly prescription of 120 mg elemental iron (n = 122) in preventing iron deficiency anemia. No significant difference was observed between the groups and the authors concluded that weekly supplementation of iron was as effective as daily supplementation in preventing anemia in pregnant women [11]. In the study by Haidar in Ethiopia, weekly iron supplementation was as effective as daily regimen in preventing anemia in breast feeding women [19]. In another study in Mexico by Casanueva and co-workers to compare the daily prescription of 60 mg elemental iron (n = 56) with weekly prescription of 120 mg elemental iron, both regimens were equally effective in preventing anemia in pregnancy. Our findings are in line with these studies [16]. In similar studies by Young and others in rural areas of Malawi and by Galloway and colleagues, 60 mg daily regimens were as effective as 120 mg weekly regimens for preventing anemia [18-20]. And similar to our study, serum hemoglobin levels were not different after 8 weeks of treatment.

In a randomized clinical trial in 2001, Goonewarden and co-workers compared the effects of daily (n = 31), three times a week (n = 35), and weekly (n = 26) prescription of 100 mg elemental iron. They measured serum hemoglobin, hematocrit, and ferritin before and after the intervention and stated that the risk of development of iron deficiency anemia was significantly higher in three times a week and weekly supplementation of iron compared with daily regimen [21]. These findings contrast with our results. The reason may be the smaller sample size, and differences in race and nutritional habits of the participants in Goonewarden's study. Mumtaz and others showed in a clinical trial that daily iron supplementation was more effective than weekly supplementation in Pakistani pregnant women. They randomly assigned 191 pregnant women with anemia (Hgb<11 g/dl) to two groups. One group received 200 mg elemental iron daily and the other group received 400 mg elemental iron twice a week. Serum hemoglobin increased significantly in women receiving daily iron supplementation. Moreover, serum ferritin was elevated only in the daily regimen [15]. The variation observed in this study can be attributed to the fact that Mumtaz and colleagues studied women who already had anemia before pregnancy. Additionally, racial, geographical, and nutritional factors could be responsible for the differences observed.

## Conclusion

Our study demonstrated all of three regimens of iron supplementation; daily, three times a week and weekly are effective for prevention of anemia during pregnancy in pregnant women without anemia. Therefore, results suggested health care providers adapt weekly regimens because of the better patients' compliance. This finding can partly be explained by the expansion of plasma volume and increased need to Iron by the fetus, which usually occur in the second trimester of pregnancy. Although iron supplementation by all three regimens could not change the level to normal, it could prevent further iron deficiency anemia.

## Competing interests

The authors declare that they have no competing interests.

## Authors' contributions

Each author has participated actively and sufficiently in this study. ZB conceived the idea and design of the study, interpretation of data, and drafted the manuscript. SHY conceived the idea and design of the study. ZB and MZ made substantial contribution to analysis and interpretation of data. MD and MM have made contribution to collecting of data and editing. Each author revised critically the manuscript and provided final approval of the version to be published.

## References

[B1] Hallberge L Fomon SJ, Zlotkin S Iron balance in pregnancy and lactation Nutrition Anemias. Nestle Nutrition Workshop Series 1992 New York. Raven Press 13 28

[B2] Bothwell TH Iron requirements in pregnancy and strategies to meet them Am J Clin Nur 2000 72 257 264 10.1093/ajcn/72.1.257S 10871591

[B3] Mukhopadhyay A Bhatla N Kriplani A Pandey RM Saxena R Daily versus intermittent iron supplementation in pregnant women Hematological and pregnancy outcome. J Obstet Gynaecol Res 2004 30 409 417 10.1111/j.1447-0756.2004.00223.x 15566454

[B4] Schaefer RM Huch R Krafft A Current recommendations for the treatment of iron deficiency anemia Rev Med Suisse 2007 3 784 800 17514929

[B5] Breymann Christian Iron Deficiency and Anaemia in pregnancy : modern aspects of diagnosis and therapy Blood cells molecules and disease 2002 29 3 506 515 10.1006/bcmd.2002.0597 12547241

[B6] Viteri FE Liu XN Martin A Tolomeik True absorption and retention of supplemental iron is more efficient when administered every-three- days rather than daily to iron- normal and iron-dedicient rats J Nutr 1995 125 82 91 7815180 10.1093/jn/125.1.82

[B7] Solomens NM Weekly versus daily oral iron administration Nutr Rev 1995 53 326 327 8643215 10.1111/j.1753-4887.1995.tb01487.x

[B8] Frazer DM Anderson GJ The orchestration of body iron intake: How and where do enterocytes receive their cues? Blood cells Mol Dis 2003 30 288 297 10.1016/S1079-9796(03)00039-1 12737947

[B9] O Neil-Cutting MA Crosby WH Blocking of iron absorption by a preliminary oral dose of iron Arch Intern Med 1987 147 489 491 10.1001/archinte.147.3.489 3827425

[B10] Bread JL Effectiveness and strategies of iron supplementation during pregnancy Am J Nutr 2000 71 1288 1294 10.1093/ajcn/71.5.1288s 10799404

[B11] Muslimatum S Schmidt MK Schultink W West CE Hautvast JA Cross R Muhilal Weekly supplementation with iron and vitamin A during pregnancy increases hemoglobin concentration but decreases serum ferritin concentration in Indonesian pregnant women J Nutr 2001 131 85 90 11303488 10.1093/jn/131.1.85

[B12] Ridwan E Schultink W Dillon D Gross R Effects of weeks iron supplementation on pregnant Indonesian women are similar to those of daily supplementation Amyclin Nutr 1996 63 884 890 10.1093/ajcn/63.6.884 8644682

[B13] Berger J Aguayo VM Tellez W Lujan C Traissac P San Miguel JL Weekly iron supplementation is as effective as 5 day per week iron supplementation in Bolivian school children living at high altitude Eur J Clin Nutr 1997 51 381 386 10.1038/sj.ejcn.1600418 9192196

[B14] Schultink W Gross R Use of daily compared with weekly iron supplementation JClin Nutr 1999 69 739 742 10.1093/ajcn/69.4.739 10197578

[B15] Mumtaz Z Shahab S Butt N Rab MA Demuynck A Daily iron supplementation is more effective than twice weekly iron supplementation in pregnant women in Pakistan in a randomized double-blind clinical trial J Nutr 2000 130 2697 2702 11053509 10.1093/jn/130.11.2697

[B16] Casanueva E Viteri FE Mares-Galindo M Meza-camacho C Loria A Schnaas L Valdes-Ramos R Weekly iron as a safe alternative to daily supplementation for noanemic pregnant women Arch med Res 2006 37 674 820 10.1016/j.arcmed.2005.11.011 16740440

[B17] Liu XN Zhang JL Yen HL Viteri FE Haemoglobin and serum feritin levels in pregnant Chinese women in response to weekly iron supplements(Abstract) proceeding of 7th Asian Congress on Nutrition; October7-11.1995, Beijing, China

[B18] Young MW Lupafya E Kapenda E Bobrow EA The effectiveness of weekly iron supplementation in pregnant women of rural northern Malawi Trop Doct 2000 30 84 88 10842553 10.1177/004947550003000210

[B19] Haidar J Omwega AM Muroki NM Ayana G Daily versus weekly iron supplementation and prevention of iron deficiency anaemia in lactating women East Afr Med J 2003 80 11 16 12755236 10.4314/eamj.v80i1.8661

[B20] Galloway R Mc Guire J Daily versus weekly: How many iron pills do Pregnant women need? Nutr Rev 1996 54 318 323 9063022 10.1111/j.1753-4887.1996.tb03795.x

[B21] Goone Wardene M Liyanage C Fernando R Intermittent oral iron supplementation during pregnancy Ceylon Med J 2001 46 132 135 12164031 10.4038/cmj.v46i4.6440

